# Assessment of vulnerability dimensions considering Family History and environmental interplay in Autism Spectrum Disorder

**DOI:** 10.1186/s12888-023-04747-3

**Published:** 2023-04-14

**Authors:** Anita Brito, Felipe Franco, Helena Brentani, Patrícia Cristina Baleeiro Beltrão-Braga

**Affiliations:** 1 Department of Microbiology, Institute of Biomedical Sciences, University of São Paulo, São Paulo, SP Brazil; 2 Present Address: Scientific Platform Pasteur-USP, São Paulo, SP Brazil; 3 Present Address: Psychiatry Institute, University of São Paulo’s Faculty of Medicine (IPq-FMUSP), São Paulo, SP Brazil; 4 Interunit Postgraduate Program On Bioinformatics, Institute of Mathematics and Statistics (IME), University of São Paulo, São Paulo, SP Brazil

**Keywords:** Autism, Genetic, Family history, Environmental factor, Autism spectrum condition, Autism Spectrum Disorder

## Abstract

**Background:**

Despite previous studies have recently shown Autism Spectrum Disorders (ASD) as having a strong genetics background, over a minimum environmental background, no study up to date has investigated the interplay between genetics and environment.

**Methods:**

We have collected data regarding Family History (FH) and Environmental Factors (EF) from 2,141 individuals with ASD and their caretakers throughout Brazil, based on an online questionnaire. Most of the ASD individuals were males (81%) and the average age was 02 years minimum for males and females, and the maximum age was 41 years for males and 54 for females. People from all states in Brazil have answered the questionnaire. Genetic inheritance was obtained based on the declared FH of Psychiatric and Neurological diagnosis. As for EF, exposure to risk factors during pregnancy was considered, like infections, diabetes, drugs/chemicals exposure, socioeconomic, and psychological factors. Respondents were invited to answer the questionnaire in lectures given throughout Brazil, and by the social networks of the NGO “The Tooth Fairy Project”. A Multiple Correspondence Analysis (MCA) was conducted to search vulnerability dimensions, and a Cluster Analysis was conducted to classify and identify the subgroups.

**Results:**

Regarding EF, social and psychological exposures contributed to the first two dimensions. Concerning FH, the first dimension represented psychiatric FH, while the second represented neurological FH. When analyzed together, EF and FH contributed to two new dimensions: 1. psychiatric FH, and 2. a psychosocial component. Using Cluster Analysis, it was not possible to isolate subgroups by genetic vulnerability or environmental exposure. Instead, a gradient of psychiatric FH with similar contributions of EF was observed.

**Conclusion:**

In this study, it was not possible to isolate groups of patients that correspond to only one component, but rather a continuum with different compositions of genetic and environmental interplay.

**Supplementary Information:**

The online version contains supplementary material available at 10.1186/s12888-023-04747-3.

## Background

Autism Spectrum Disorder (ASD) is considered a Neurodevelopmental Disorder (NDD) with high prevalence, characterized by deficits in social communication, as well as restricted and repetitive behaviors. One in 44 children aged 08 years old has been diagnosed with ASD in the United States [1, 2]. Important to point out that ASD prevalence is underestimated and not well explored in Brazil. There is only one outdated study [3] that assessed children from only one town in the Southeast region of Brazil in 2011, estimating 27.2/10,000 in a population of 198,2 million, which is far from 1%-2% estimated in the United States [2]. Nowadays, considering the 208 million inhabitants, this number should be between 2–4 million autistic people in Brazil.

ASD genetic architecture is complex and it is associated with a higher burden of very rare and/or de novo deleterious variations, like SNVs (Single Nucleotide Variations) and CNVs (Copy Number Variations) [4–8]. On the other hand, more than 50% of ASD vulnerability is assigned to the additive effect of common variation, creating a polygenic risk score (PRS) [9–11], and an additive effect of rare and common variations have also been described [12]. It is important to note that the genetic component mainly represented by PRS has been characterized as a genetic psychiatric vulnerability, corroborating the presence of different psychiatric conditions in ASD families [13, 14].

The epidemiological or Genome-Wide Association Study (GWAS) based ASD heritability studies reinforce the predominant effect of genetic components [15, 16], but they do not rule out the contribution of Environmental Factors (EF). The environment, in which the mother has been exposed during gestation, may contribute to a disturbance in fetal neurodevelopment, thus increasing the chances of developing ASD in many different ways, like the fetus being exposed to biological components, being affected by metabolic and/or inflammatory diseases (diabetes, eclampsia, infection, gain of weight), or even being exposed to chemicals (pollution, legal or illegal drugs, pesticides, heavy metals) [17, 18]. Also, different factors associated with emotional stress, including the mother's psychopathology (depression, anxiety), socioeconomic factors, aggression (physical, sexual, or emotional), and stressful situations (losing a job, death of a loved one, violent neighborhood) have been associated with neurodevelopmental trajectories [19–21].

Most epidemiological studies looking for ASD association with environmental exposition search for one exposure factor at a time. However, it is important to consider that individuals, especially in developing countries, are not usually exposed to only one factor, but different correlated exposures at the same time [22]. In a previous study, with 67 ASD cases, we showed that prenatal vulnerability components and epigenetic age acceleration as a proxy of postnatal stress exposure were associated with ASD severity and functionality scores measured at 08 years of age [23]. We used psychiatric FH representing genetic vulnerability and gestational exposure to EF to search the prenatal vulnerability components using principal component analysis (PCA).

It is important to note that deleterious variations associated with ASD have been described not only in other psychiatric disorders, but also in neurological conditions, such as epilepsy or intellectual deficiencies [4–8]. Autistic individuals present a vast clinical heterogeneity and may present several comorbidities, including other psychiatric disorders but also, neurological diseases, and genetic syndromes [8, 24–26]. In this paper, to better explore prenatal vulnerability components and search possible ASD subgroups, according to their vulnerability components, we used a larger sample size including individuals from all regions in Brazil. A broader questionnaire was used considering different questions to more reliably assess gestational exposure to EF, and included neurological and intellectual disability FH to assess genetic vulnerability. Our aim was to better explore vulnerability components and search possible ASD subgroups according to their vulnerability components.

## Methods

### Research ethics, patient consent, and procedures of data collection

This project was approved by the Ethical Committee from CAAE 61,093,416.0.00005467. The patients or their legal guardians have signed the consent form.

To better understand neuropsychiatric FH (as a proxy of the polygenic contribution) and EF contribution to ASD, in this work, we carried out an exploratory analysis through MCA, which reduces the dimensionality of categorical datasets, providing new latent variables, which makes it possible to estimate the contributions of each initial variable to the new ones [27]. Before performing MCA with EF and FH, to better evaluate gestation exposure to EF, we first performed the MCA considering different types of environmental exposure such as socioeconomic, psychological, chemical exposure, and gestational problems. We performed the MCA using only FH to explore psychiatry and/or neurological familial contributions. We also explored through a cluster analysis, using the component scores, subgroups of patients by different genetic backgrounds and EFs.

From October 2018, till December 2019, families of ASD individuals were recruited during ASD conferences held throughout Brazil, via social networks, and via email, being the last one directed to families who had previously donated deciduous teeth of ASD children to "The Tooth Fairy Project''. Independent adults with ASD, or first-degree family members of ASD-dependent individuals, were invited to fill out the questionnaire. Simplex and multiplex families, with males or females with an ASD diagnosis, were accepted.

Since our research was conducted based on answers obtained from a questionnaire, four different questions were designed in order to certify the ASD diagnosis reported by respondents:

- Has the ASD person been diagnosed by a healthcare professional?
  Possible answers: Yes - No - I don't know
- If you already have a diagnostic report, could you send it to our team? If so, send it to our email.
  Possible answers: Yes - Not yet - I don´t know
- What is the specialty of the person who diagnosed the person with ASD?
  Possible answers: Psychiatry - Neurologist - Pediatrician - Psychologist - Multi professional team - I don't know
- What is the name of the professional or service that gave the diagnosis?
  Possible answers: (full name) - I don't know.

Based on the responses, five classes were created, considering the diagnosis given and which information was provided for its reliability: ASD individuals Diagnosed by a Health Professional, with a Report (ASDHPR); ASD individuals Diagnosed by a health professional, but the Report was Not Available (ASDDRNA); ASD individuals diagnosed by a health professional that Will Send a Report (ASDWSR); ASD individuals Auto Reported (ASDAR); and ASD individuals diagnosed by a Health Professional, but Without a Report (ASDHPWR). Individuals with responses "I don’t know” in any of the above questions were excluded.

### Questionnaire

The questionnaire has 132 questions and it was divided into 08 sections. The questions were mostly designed as multiple-choice, checkboxes, dropdown, linear scale, multiple-choice grid, and checkbox grid questions. In order to fill out names and addresses, or to complement any answer the respondents felt necessary, short or long paragraphs were used. The respondent was not able to go to the next page without answering all questions presented in each section, avoiding missing data.

Important to point out that the timestamp for each answer was verified in order to detect that bots were not responding to the questionnaire. The short and long answers, like the name of the autistic person, name of their parents, their weight when they were born, mother's weight before and after the pregnancy, their height, addresses, emails and telephone numbers of the parents, name of medications, name of the doctors, as well as answers about any other relevant information they would like to share were verified and all set of answers are different from each other.

In order to obtain ASD severity of each respondent, they must answer a question about the level of autism, which explained the levels as Level 1 (requiring support), Level 2 (requiring substantial support), and Level 3 (requiring very substantial support). The possible answers are: Level 1, Level 2, Level 3 and I don't know.

The questions designed to gather information about EF, like fetal environment/gestational problems (obesity, hypertension, diabetes, infections), toxic exposure (chemicals, alcohol and/or marijuana abuse, active or passive smoking), and psychosocial stress (aggression, mother depression, stressing situations, mother/father education, and house income), to which mothers could have been exposed during pregnancy, were defined according to scientific literature [17–21].

FH of a psychiatric or neurological specific condition (ASD, Alzheimer's, Parkinson, Schizophrenia, Epilepsy, Tics, Down Syndrome, Bipolar Disorder, Intellectual Disability, Depression, Hyperactivity, Attention Deficit Disorder, OCD, Panic Disorder, and Anxiety Disorder) was considered from the primary and secondary kinship (parents, uncles and aunties, grandparents, first cousins), based on the presence or not of the condition in any consanguineal relative in the list during any lifetime.

### Analysis

Analyses were conducted in RStudio software, version 3.6.1 [28], with the following packages: ggplot2 [29], factoMineR [30], and factoExtra [31]. When necessary, all questions were converted in order to have responses in the same direction from better to worse scenarios. In order to guarantee that the same content was covered, we only considered individuals with answers without “I don’t know” to form each score. Then, composite scores were categorized. It is important to point out that they are categorized according to a severity gradient, with 0 meaning "No problem" and the highest number representing the worst case. For example, to assess the exposure "Depression Symptoms", we have asked seven different questions, asking about the presence, or not, of a specific symptom. For each individual, we summed up the number of presented symptoms. Then, we categorized the levels of depression based on the number of symptoms. As most of the respondents answered “I don't know” to the questions related to chemical exposure, we did not use chemical exposure in further analysis. All other variables were formed as described in supplemental methods. Supplemental Table 1 shows the number of questions and the content of different questions used to compose different environmental exposure scores and dismissed individuals. The histogram of all EF and FH levels can be seen in Supplemental Figures 1 and 2.

Since the prevalence of ASD is different between the biological sexes [1, 2], a test of association between sex and the 23 variables considered in the study was carried out. In order to analyze these variables, a chi-square (χ2)test was employed on the contingency tables. *P*-values < 0.05 were considered significant.

#### MCA

We used MCA to analyze the marginal total values ​​of the row and column, and also to calculate the expected value. Thus, all variables were standardized by χ2 and we sought similar occurrences of response. We used the standard recommendations from the factoMineR package. The MCA graphics were built using the factoExtra package. In the MCA, three analyses were considered: 1—EF; 2—FH; 3—EF with FH. First, we calculated the eigenvalue or inertia, percentage of explained variance, and percentage of accumulated variance. Then, variable contribution (or absolute contribution) and cosine^2^ (or relative contribution) were calculated for each dimension. We explored the first two dimensions and, to define which variables are the most important to each dimension, we considered variables with contributions higher than the average of all variables.

### Group analysis

In the cluster analysis, the “HCPC” function, from the “FactoMineR” package, was used, adopting Ward's method to perform the hierarchical clustering. The input data were derived from the MCA, considering EF and FH. For calculation, the Euclidean distance was adopted and the threshold for the number of groups was determined by partition considering the one with the higher relative loss of inertia. After defining the groups, a graph was built containing a biplot representation of the MCA with the individuals and the resulting cluster.

## Results

In this study, 2,141 ASD individuals were assessed by an online questionnaire. Out of 2,141 questionnaires (0.3%) were answered by ASD cases and (99.7%) were answered by their caretakers. Most of the ASD individuals were males (81%). About one percent of the patients were adopted, so they were dismissed from our FH analyses. However, they were considered in the sample characterization (sex, place of birth, average age, level of autism, and age of diagnosis). The average age of the ASD individuals was 02 years minimum for males and females, and the maximum age was 41 years for males (average of 11.5 years old) and 54 for females (average of 13.5 years old). The majority of our sample was diagnosed between 01 and 04 years old (74%), and the respondents reported that 47% fall into Level 1 of ASD severity. People from all states in Brazil have participated in this study, being the greater concentration of respondents in the Southeast region (the financial center of Brazil) representing 54.3% of the total sample (Table 1).

**Table 1:**
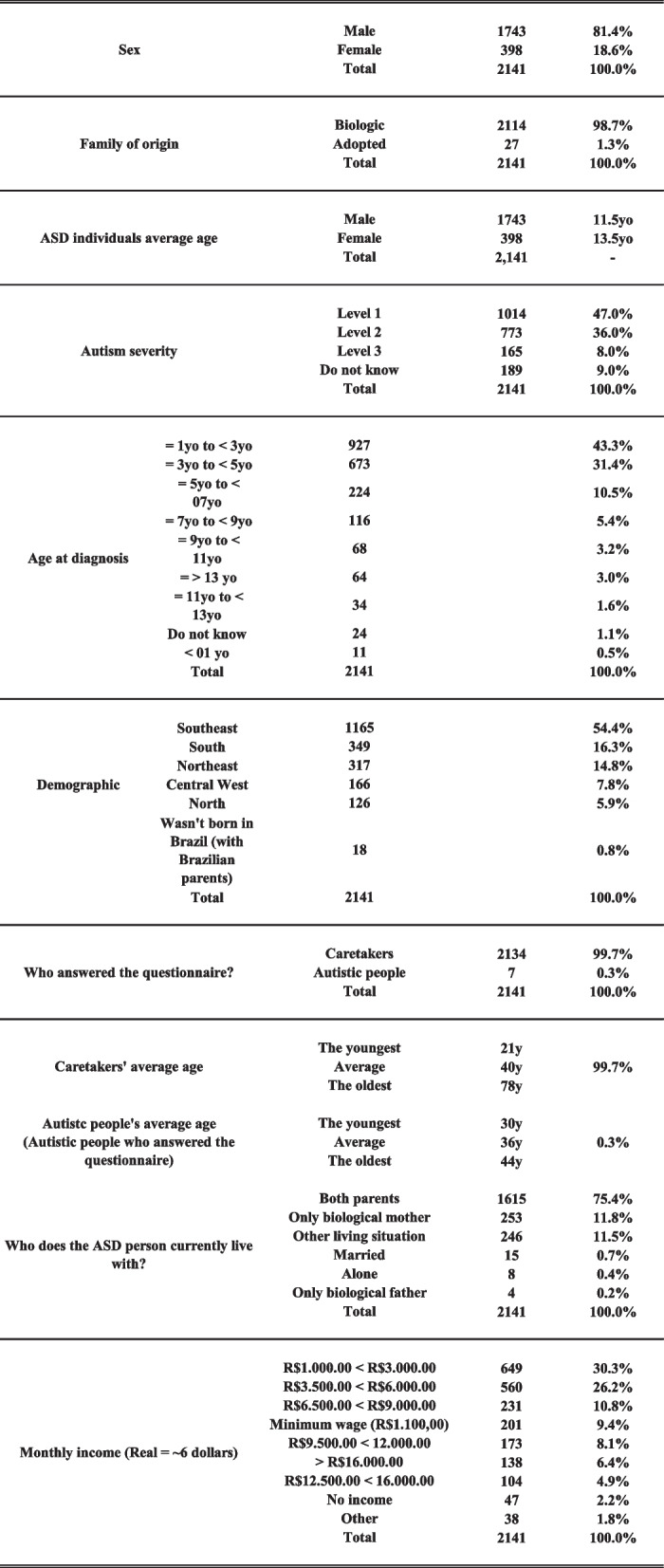
Sample description (*n* = 2,141)

Table 2 shows the number and percentage of individuals in each ASD diagnosis category, the Average Age of the proband when the questionnaire was filled out, as well as Standard Deviation (SD). Additionally, 10% of ASDHPR sent a report confirming the ASD diagnosis.

**Table 2:** Diagnosis assessment

**Category**	**Number of participants**	**%**	**Average age**	**SD**
**ASDHPR**	**1501**	**70.1%**	**8.9 yo**	**5.7**
**ASDDRNA**	**297**	**13.8%**	**9.8 yo**	**6.8**
**ASDWSR**	**261**	**12.2%**	**8.2 yo**	**7.4**
**ASDAR**	**12**	**0.6%**	**7.8 yo**	**4.9**
**ASDHPWR**	**70**	**3.3%**	**10.7 yo**	**9**
**TOTAL**	**2141**	**100**	**9.1 yo**	**6.7**

*1501 ASDHPR* ASD Individuals diagnosed by a health professional, with a report, *297 ASDDRNA* ASD Individuals diagnosed by a health professional, but the report was not available, *261 ASDWSR* ASD Individuals diagnosed by a health professional that will send a report, *12 ASDAR* ASD Individuals auto reported, and *70 ASDHPWR* ASD Individuals diagnosed by a health professional, but without a report, *Average age* of the proband when the questionnaire was filled, *SD* Standard deviation

As explained in the Methods section, the number of respondents could be different for each analysis, depending on whether any invalid answer (“I don´t know”) was given to form the exposure score variable. Therefore, from 2,141 respondents, 359 were dismissed from the EF analysis, and 1,782 respondents were considered to search gestational environmental exposure components for ASD. As for FH analysis, 1,237 respondents were dismissed, being considered 904 respondents in our final sample (Supplemental Figure 3). Before performing the MCA analysis, we searched for differences in the gestational environmental and FH scores between males and females (Supplemental Table 2). Interestingly, there was a lower relative occurrence of drug use during pregnancy in female individuals (chi-value = 7.89, *p*-value = 0.019), and a higher relative occurrence of FH for Panic in female individuals (chi-value = 5.76, *p*-value = 0.016).

**Fig. 1:**
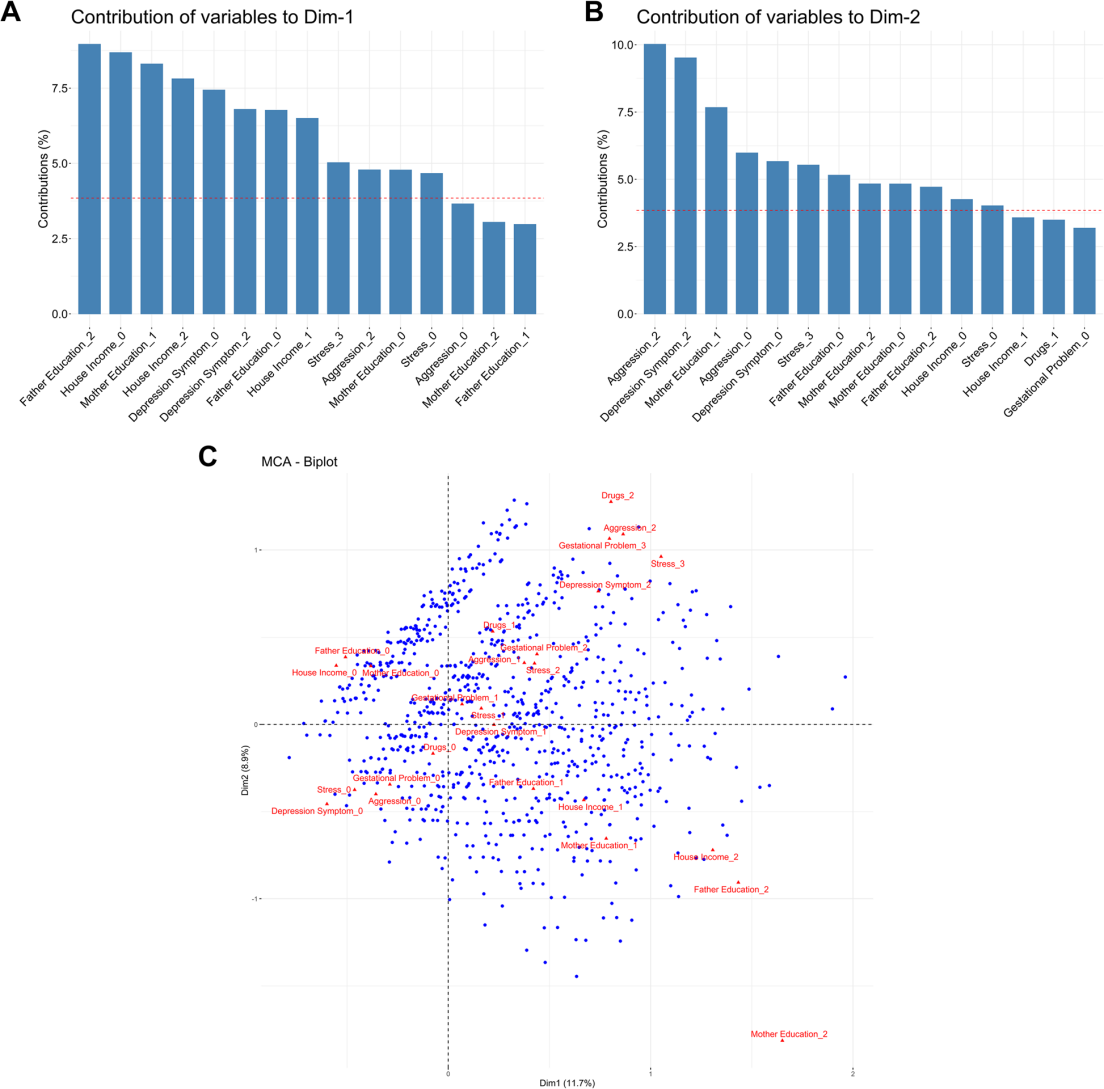
MCA with Environmental Factors variables (1782 individuals and 8 variables). **A** Barplot of contribution of variables to first dimension. **B** Barplot of contribution of variables to second dimension. **C** MCA biplot visualization of response levels

Assuming that individuals are not exposed to just one EF, and to better evaluate environmental exposure components/dimensions, we first performed the MCA considering the different types of environmental exposure scores, such as aggression, exposure to legal and illegal drugs, mother depression, stressful situations, and gestational problems (Fig. 1A, B and C). In order to improve comprehension, hereafter we will refer to components/dimensions only as dimensions in the MCA analysis. The eigenvalues and proportion of explained inertia of the first ten dimensions can be observed in Supplemental Table 3. The explained variance (inertia) for the two main dimensions formed in the MCA for EF was 11.73% and 8.92%, for the first and second dimensions, respectively. We can observe that for each dimension, considering as important features, the ones with a contribution value above all factors on average, Father and Mother Education, House Income, Depression Symptoms, Stress, and Aggression contribute to dimensions 1, 2, and 3. Considering only the 2 first dimensions, education and house income are more important to dimension 1, whereas high levels of Depression Symptoms and aggression during gestation are more important to dimension 2. Dimension 1 could be characterized by a gradient of social vulnerability, and dimension 2 represents a gradient of psychological stress vulnerability. Biological exposures represented by gestational problems and drugs contributed after the 4th dimension (Supplemental Table 4).

**Fig. 2:**
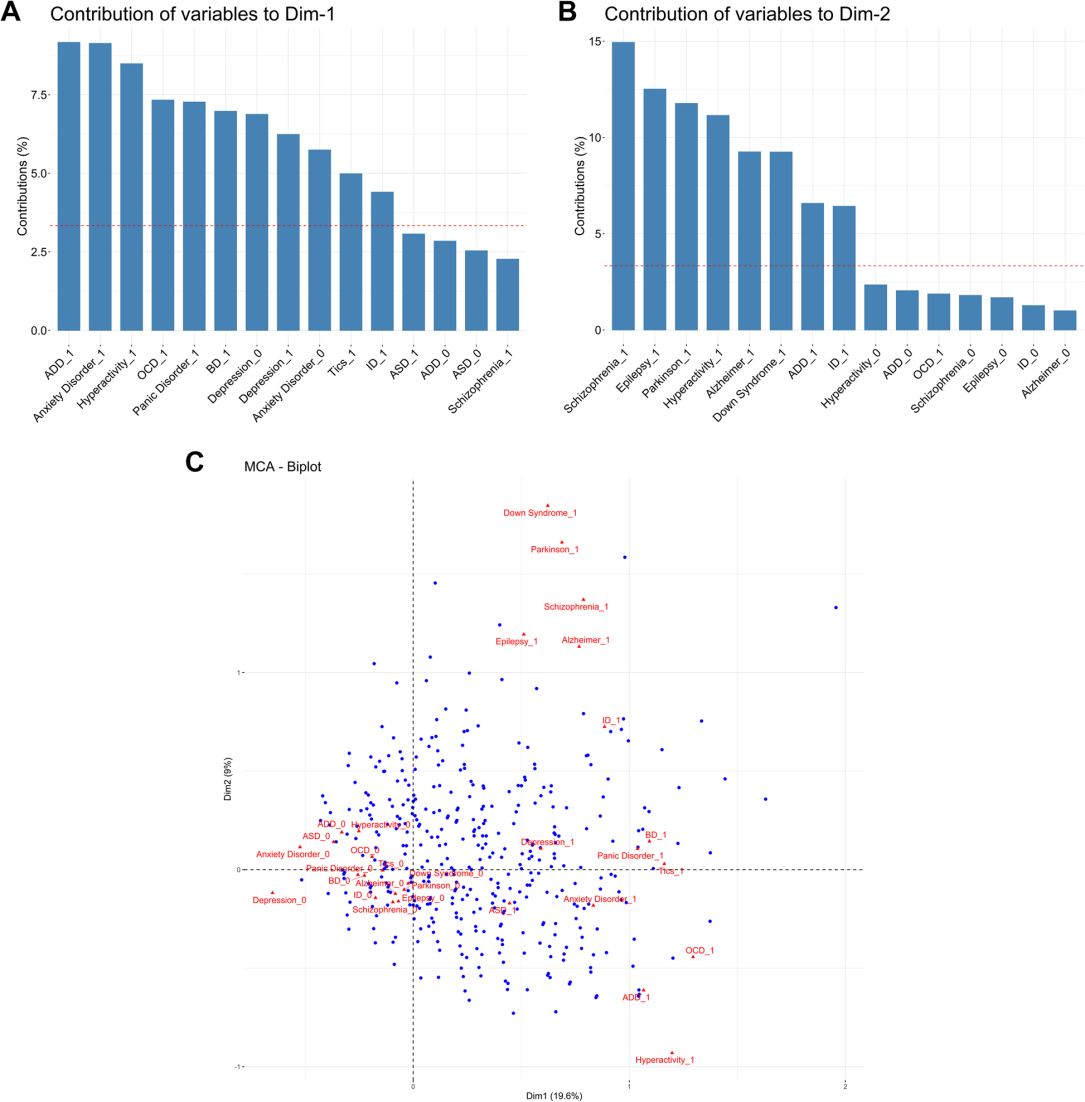
MCA with FH variables (904 individuals and 15 variables). **A** Barplot of contribution of variables to first dimension. **B** Barplot of contribution of variables to second dimension. **C** MCA biplot visualization of response levels. Autism Spectrum Disorder (ASD); Bipolar Disorder (BD); Attention Deficit Disorder (ADD); Obsessive Compulsive Disorder (OCD); Intellectual Disability (ID)

Considering FH, we asked about psychiatric disorders and neurological diseases, including neurodevelopmental and neurodegenerative disorders. Then, we performed an MCA considering only FH to explore associations within familial scores. The eigenvalues and proportion of explained inertia of the first ten dimensions can be observed in Supplemental Table 4. The explained variance for the two main dimensions formed in the MCA for FH was 19.58% and 8.98%, for the first and second dimensions, respectively. Figure 2 A and B show the exposure factors that pass the selected threshold to consider it significantly associated with the dimensions (Supplemental Table 4), and in Fig. 2C, we can observe a biplot of the first two dimensions. Positive FH of Hyperactivity, Attention Deficit Disorder (ADD), and Intellectual Disability (ID) have significant contributions to both first dimensions, being ADD more important to the first, and the others to the second dimension. Down Syndrome was included only in the second dimension. ASD FH did not contribute significantly to the first two dimensions, although according to the quality of representation, it will be assigned to dimension 1. Interestingly, all psychiatric disorders, but schizophrenia, contributed to dimension 1, whereas all neurological diseases contributed more to dimension 2. So, we characterized dimension 1 as psychiatric FH and dimension 2 as neurological FH.

Finally, we performed an MCA with EF and FH analyzed together, and 828 respondents, out of 2,141, were considered (Supplemental Table 5; Supplemental Figure 4A, B, and C). The eigenvalues and proportion of explained inertia of the first ten dimensions can be observed in Supplemental Table 5. The explained variance for the two main dimensions formed in the MCA for EF and FH was 9.75% and 6.35%, for the first and second dimensions, respectively. In dimension 1, most Psychiatric FH and “Mother depression during gestation" were considered significant, while in dimension 2, "Mother and Father Education" and "House Income", but also "Mother depression during gestation", as well as "Aggression" and "Stress" were significant. Gestational problems were significant only to dimension 3. In dimension 1, it is possible to notice a higher concentration of psychiatric disorders, interpreted as a polygenic risk dimension, whilst there is a higher concentration of socioemotional factors in dimension 2 (Supplemental Figure 4B), considered a socioemotional environmental dimension. On the same graphical representation, using ASD classes and sex, no differences were observed (Supplemental Figure 5).

**Fig. 3:**
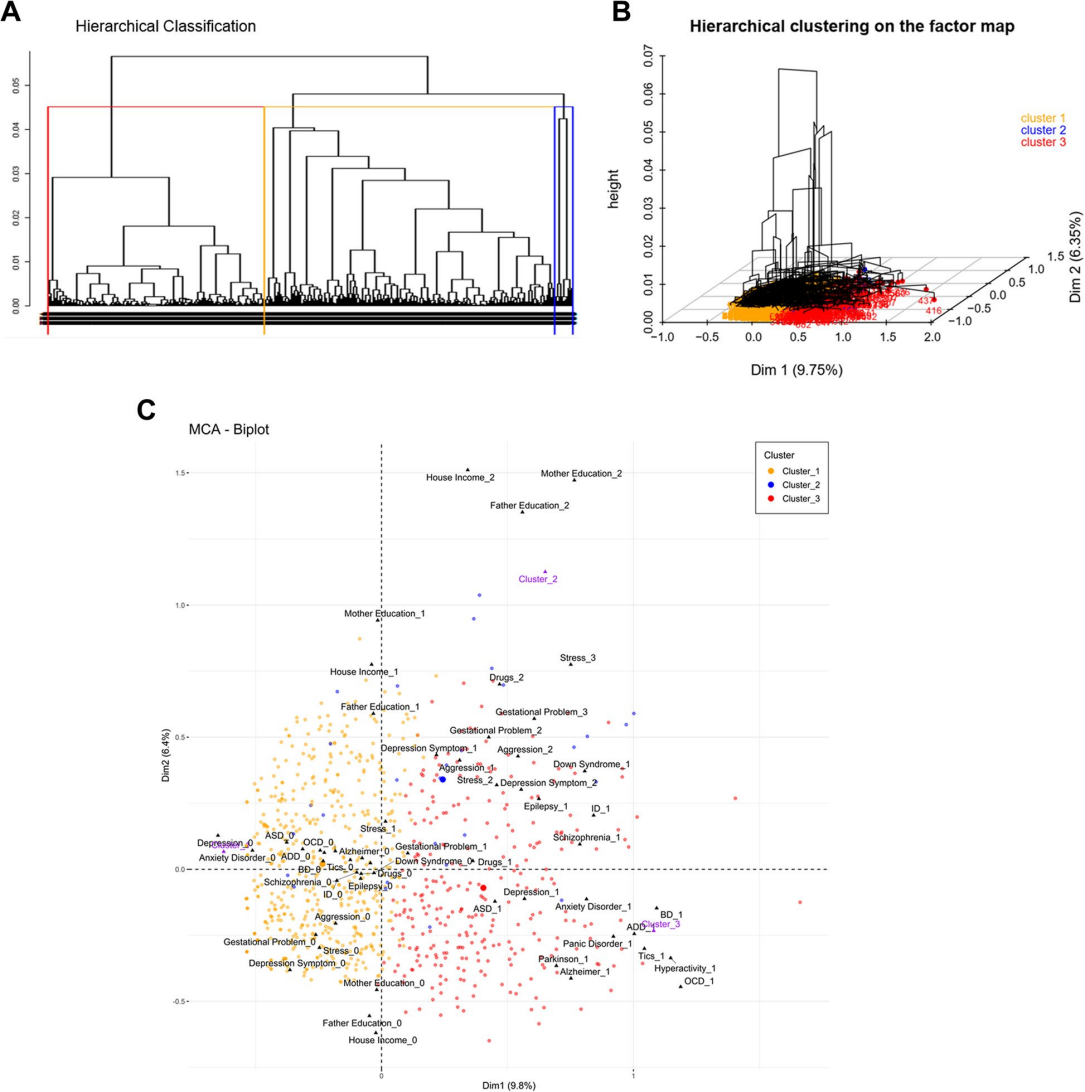
Cluster Relationship. Cluster relationship of MCA results considering EF and FH variables (828 individuals and 23 variables). Three clusters are represented in orange, blue and red. **A** Hierarchical classification. **B** Three-dimensional representation of hierarchical clustering on the factor map. **C** MCA biplot of individuals by cluster colors on the polygenic (Dim 1) and environmental (Dim 2) axes

To explore possible subgroups according to EF and FH dimensions, we performed a cluster analysis. Three clusters were observed (Figs. 3A and B). Individuals in cluster 1 have all the spectrum of environmental exposure factors best characterized by a gradient of social exposure factors, but no FH, while individuals in cluster 3 have positive FH. In Fig. 3C, we can also observe that it seems that dimension 3 (including some neurodevelopmental disorders and also gestational problems) is responsible for separating Cluster 2.

## Discussion

ASDs are complex multifactorial NDDs with distinct and still not-well-established etiologies. In this study, we tried to infer the relevance of genetic factors related to ASD using FH information reported by the respondents, in which they provided data on the history of psychiatric, neurological, and neurodevelopmental conditions of blood relatives. Similarly, we sought information on prenatal environmental exposure including biological and socio-physiological stressors. To our knowledge, there are no studies that have observed the interplay between EF along with familial contributions in such a comprehensive manner in ASD vulnerability.

Regarding our sample, the initial characterization of the studied group was done taking into consideration data such as sex, age, level of ASD, and geographical location. Our group sample points to a frequency of 04 males to 01 female (81%-19%, respectively), which agrees with scientific literature [2, 16, 32, 33]. Regarding the age of the respondents, most of them were born predominantly after the 2000s, more precisely from 2005 onwards. As for ASD levels, there was a higher occurrence of level 1 (47%) and level 2 (36%). Based on these data, it is not possible to infer whether they are the most frequent in the Brazilian population or if it is only the perspective of the respondents. Considering ASD sex differences, when we analyzed variables individually, we found significant differences in males and females for Drug Use and Panic Disorder. However, there were no differences observed in our MCA analysis (Supplemental Figure 5).

The first goal of this study was to explore how different gestational exposure to EF, such as mother depression, toxic exposure, social factors, stressful situations, and aggression, contributed to ASD environmental dimensions. Contrasts in sex, birth outcome, and fetal neurodevelopment have already been distinctively associated with latent profiles of maternal prenatal stress [34].

In our previous paper, the psychosocial component was also very important, but considering that environmental exposure (e.g., infection during gestation), maternal diabetes, obesity, and certain drugs and chemicals have strong evidence of association with ASD [8, 17, 19, 35–39], we expected that, with a larger sample size, the first dimension would be explained by those biological agents. However, the scores related to environmental exposure showed that the two first dimensions were a gradient of social vulnerability and a gradient of psychological stress. As for economic status and other social issues, pregnant women can experience unhealthy and inappropriate environments due to financial problems, which may increase vulnerability to autism in the offspring [40]. Regarding EF, father and mother education has already been suggested by other authors [19, 37], and it is sometimes used as a proxy for socioeconomic status. Socioeconomic disadvantage during pregnancy has been shown to substantially affect the development of the offspring's brain [41]. Interestingly, maternal socioeconomic problems seem to be related to reduced concentrations of the proinflammatory cytokine IL-8 during gestation. Additionally, lower levels of IL-8 were also linked to offspring’s neurologic abnormalities, independently of maternal medical history [42].

There is evidence suggesting that prenatal maternal stress may increase ASD outcome [43–46] and severity of clinical presentation [47]. Prenatal maternal psychosocial stress has been associated with inflammation in human and animal models and linked to a wide array of psychiatric and neurodevelopmental conditions in offspring, including ASD [48, 49]. Additionally, maternal immune activation has a pivotal role in ASD, [50–53]. Besides inflammation, other mechanisms such as HPA axis (Hypothalamic–pituitary–adrenal axis) disturbance, which may unbalance the mother's homeostasis, have already been associated with psychosocial stress exposure [54].

The second goal was to explore the contribution of psychiatric and neurological FH to ASD, as we have only analyzed psychiatric disorders in our small sample size previous study [23]. Our FH analysis considered cases of psychiatric disorders and neurological diseases, including neurodevelopmental and neurodegenerative forms in all respondents. Interestingly, it was possible to identify two dimensions related to ASD individuals in our studied group, one referring to more Psychiatric Disorders and the other more to Neurological diseases. GWAS-based PRS of psychiatric disorders appear more frequently correlated among them, which seem to be divergent in neurological disorders [14] supporting our findings. For almost all NDDs, FH contributed to both dimensions, but Down Syndrome. Intriguingly, Schizophrenia, which is considered a Psychiatric Disorder, and has been modeled as both neurodevelopmental and neurodegenerative [55–58], was closer to the Neurological disorders dimension. Recently, similarities in white matter deficit patterns in patients with Schizophrenia and Alzheimer's disease were observed as Schizophrenia patients have gotten older [59].

The third goal was to search ASD vulnerability dimensions considering both EF and FH. The first dimension was associated with psychiatric FH as a proxy of major polygenic contribution, in accordance with the genetic correlation between psychiatric disorders' PRS, but not neurological diseases' PRS [14]. The second major dimension was a psychosocial gradient supporting the genetic component in ASD, as well as the environmental exposure importance to ASD vulnerability.

Finally, our last goal was to explore how the dimensions related to vulnerability would contribute to forming ASD subgroups by a clustering analysis. We first hypothesized that clusters would not be clearly separated, which was later revealed in our results (Fig. 3), and different compositions of ASD vulnerability would be represented as a spectrum.

### Limitations

The diagnosis assessment was not based on a clinical evaluation made by our group, nor data collected directly from medical electronic records. However, in this work, more than one question was designed to assess the diagnosis's reliability; e.g., the name of the health professional, as well as their specialty. Ergo, more than 90% have an ASD diagnosis given by a health professional.

We had to rely on respondents' memories to provide answers regarding pregnancy time and possible exposure factors the mothers had been exposed to during that specific pregnancy. Besides, autistic people who responded to the questionnaire (0.3%) may have had difficulties gathering some information regarding their mother's pregnancy, although we have not received any complaints. It is known that one can have false or blurred memories, mainly if the mother has had more than one pregnancy and if it was over five (or more) years ago.

Another limitation is that FH was considered only through Neurological and Psychiatric data presented by respondents, as genetic tests would not be possible at this first moment in our study. Nevertheless, our intention at this first moment was to compare the presence of ASD and other Neurological or Psychiatric conditions along with EF. Genetic tests are being considered for our next steps.

Unfortunately, in our work, we couldn't observe differences between males and females when considering all variables together, only when we considered each variable individually. Although we had 2,141 participants, most of them were males (81% males and 19% females), which could limit a comprehensive analysis based on sex differences.

Important to point out that some respondents have answered "I don't know", hence they were excluded from the whole analysis. As we did not test differences between included and excluded respondents, this could be a limitation in our work.

Finally, we have faced a few problems regarding collecting data. Even though collecting data online is much more practical than our previous method, in which people had to fill out paper forms and send them to our lab via post, we have received emails or messages through our social media saying that people could not fill out the online form due to bad Internet connection. In a few cases, the answer was duplicated, as one pressed the button "Send" more than once due to Internet lag. The duplicated answers were discharged. An app, or another platform, might be considered in order to achieve better results. Even though we have reached over two thousand people throughout the whole country, we feel that improving data collection should be of great value to those who wanted to participate, but could not. Still, as an exploratory study, it contributed to a better understanding of the possible interplay between genetics and EF in ASD.

### Future directions

Future research is highly encouraged in other populations, as our results were very intriguing, raising questions on the importance of looking at the individuals and the interplay between genetics and the environment. Also, the inclusion of other questions about social support could be considered, as well as positive environmental exposure, such as enriched environments. We intend to continue this study by applying genetic tests on part of the participants to establish genotypes and phenotypes, as well as to check possible genetic overlap among neurological and psychiatric diseases in FH, using different analysis techniques such as factor analysis and SEM to search for vulnerability components.

### Community involvement

This work was possible due to the involvement of parents/caregivers of autistic people as well as the active participation of adult-autistic people. Community service providers such as Biologists, Neuroscientists, Psychologists, and Psychiatrists were directly involved in helping to elaborate the questionnaire in a way to be practical and understandable. Of note, all professionals involved in this search are directly involved in treating and searching for autism, hence, their expertise and helpful input reflected their professional experiences rather than lived experience.

## Conclusion

In our study, genetics and EF are present in the population studied and were considered as two different dimensions. The first dimension is the FH, confirming the importance of genetics. However, EF, which represents the second component, may be more important than previously reported by Bai and colleagues (2019) [16]. Additionally, it was not possible to separate subgroups by genetic or environmental dimensions, but rather a continuum where an EF and FH interplay was observed.

## Supplementary Information

**Additional file 1:** **Supplemental Figure 1**. Risk Factors Distribution. Distribution of response levels for risk factors variables. The numbers of individuals (n) are displayed in the respective graphs. **Supplemental Figure 2**. Family History Distribution. Distribution of response levels for family history variables. In all graphs, the number of individuals (n) was 904. Autism Spectrum Disorder (ASD); Attention Deficit Disorder (ADD); Obsessive Compulsive Disorder (OCD). **Supplemental Figure 3**. Flowchart. Flowchart of the methodological approach. Autism Spectrum Disorder (ASD); Bipolar Disorder (BD); Attention Deficit Disorder (ADD); Intellectual Disability (ID); Obsessive Compulsive Disorder (OCD).** Supplemental Figure 4**. MCA with risk factors and family history variables (828 individuals and 23 variables). A,  Barplot of contribution of variables to first dimension; B, Barplot of contribution of variables to second dimension; C, MCA biplot visualization of response levels. Autism Spectrum Disorder (ASD); Bipolar Disorder (BD); Attention Deficit Disorder (ADD); Obsessive Compulsive Disorder (OCD); Intellectual Disability (ID). **Supplemental Figure 5**. MCA biplot of individuals by sex considering EF and FH variables. Males and females are represented by blue and red, respectively. Abbreviations: ASD: Autism Spectrum Disorder; BD, Bipolar Disorder; ADD, Attention Deficit Disorder; OCD, Obsessive Compulsive Disorder; ID, Intellectual Disability. **Supplemental Table 1**. Number of questions and the content of different questions used to compose different environmental exposure scores and dismissed individuals. **Supplemental** **Table 2**. Gestational environmental and FH scores between males and females. Results of the association test between biological sex versus environmental factors and Family History variables. Abbreviations: ASD: Autism Spectrum Disorder; BD, Bipolar Disorder; ADD, Attention Deficit Disorder; OCD, Obsessive Compulsive Disorder; ID, Intellectual Disability. **Supplemental** **Table 3**. Eigenvalues, Inertia, Contributions and Cos^2^of Risk Factors. Risk factors (1782 individuals and 8 variables) - The eigenvalues and proportion of explained inertia of the first ten dimensions, contributions for the variables and squared cosines. Bold characters correspond to positive characteristics. **Supplemental** **Table 4**. Eigenvalues, Inertia, Contributions and Cos^2^ of Family History. Family history for psychiatric disorders - The eigenvalues and proportion of explained inertia of the first ten dimensions, contributions for the variables and squared cosines. Bold characters correspond to positive characteristics. Autism Spectrum Disorder (ASD); Bipolar Disorder (BD); Attention Deficit Disorder (ADD); Obsessive Compulsive Disorder (OCD); Intellectual Disability (ID). **Supplemental** **Table 5**. Eigenvalues, Inertia, Contributions and Cos^2^ of Risk Factors and Family History. Risk factors and family history for psychiatric disorders - The eigenvalues and proportion of explained inertia of the first ten dimensions, contributions for the variables and squared cosines. Bold characters correspond to positive characteristics. Autism Spectrum Disorder (ASD); Bipolar Disorder (BD); Attention Deficit Disorder (ADD); Obsessive Compulsive Disorder (OCD); Intellectual Disability (ID).

## Data Availability

The datasets generated and/or analyzed during the current study are not publicly available due to exposition of respondents’ data, such as name and other private information, but are available from the corresponding author on reasonable request.

## Abbreviations

ASC: Autism Spectrum Condition
ASD: Autism Spectrum Disorder
EF: Environmental Factor
FH: Family History
GWAS: Genome-Wide Association Study
HPA axis: Hypothalamic–Pituitary–Adrenal axis
MCA: Multiple Correspondence Analysis
