# Supplementary material for: Assessment of vulnerability dimensions considering Family History and environmental interplay in Autism Spectrum Disorder
Source: BMC Psychiatry. 2023 Apr 14;23:254. doi: 10.1186/s12888-023-04747-3 (PMC10105456; doi:10.1186/s12888-023-04747-3)
Supplement: Supplementary file 1 — Additional file 1: Supplemental Figure 1. Risk Factors Distribution. Distribution of response levels for risk factors variables. The numbers of individuals (n) are displayed in the respective graphs. Supplemental Figure 2. Family History Distribution. Distribution of response levels for family history variables. In all graphs, the number of individuals (n) was 904. Autism Spectrum Disorder (ASD); Attention Deficit Disorder (ADD); Obsessive Compulsive Disorder (OCD). Supplemental Figure 3. Flowchart. Flowchart of the methodological approach. Autism Spectrum Disorder (ASD); Bipolar Disorder (BD); Attention Deficit Disorder (ADD); Intellectual Disability (ID); Obsessive Compulsive Disorder (OCD). Supplemental Figure 4. MCA with risk factors and family history variables (828 individuals and 23 variables). A, Barplot of contribution of variables to first dimension; B, Barplot of contribution of variables to second dimension; C, MCA biplot visualization of response levels. Autism Spectrum Disorder (ASD); Bipolar Disorder (BD); Attention Deficit Disorder (ADD); Obsessive Compulsive Disorder (OCD); Intellectual Disability (ID). Supplemental Figure 5. MCA biplot of individuals by sex considering EF and FH variables. Males and females are represented by blue and red, respectively. Abbreviations: ASD: Autism Spectrum Disorder; BD, Bipolar Disorder; ADD, Attention Deficit Disorder; OCD, Obsessive Compulsive Disorder; ID, Intellectual Disability. Supplemental Table 1. Number of questions and the content of different questions used to compose different environmental exposure scores and dismissed individuals. Supplemental Table 2. Gestational environmental and FH scores between males and females. Results of the association test between biological sex versus environmental factors and Family History variables. Abbreviations: ASD: Autism Spectrum Disorder; BD, Bipolar Disorder; ADD, Attention Deficit Disorder; OCD, Obsessive Compulsive Disorder; ID, Intellectual Disability. Supplemen [file 12888_2023_4747_MOESM1_ESM.docx]

**Supplemental material**

**Methods**

**Analysis**

The variables were considered as the following for score's value before conducting the analysis in RStudio software.

- **Depression Symptom (levels 0, 1 and 2):**

if (Sadness >= 1) Sadness = 1

if (Anxiety >= 1) Anxiety = 1

if (Weeping >= 1) Weeping = 1

if (Demotivation >= 1) Demotivation = 1

if (Irritability >= 1) Irritability = 1

if (Guilty >= 1) Guilty = 1

if (Suicidal Ideation >= 1) Suicidal Ideation = 1

Depression Symptom = Σ (all symptoms)

if (Depression Symptom <= 1) Depression Symptom = 0

if (Depression Symptom >= 2 AND <= 4) Depression Symptom = 1

if (Depression Symptom >= 5) Depression Symptom = 2

- **Gestational Problem (levels 0, 1, 2 and 3):**

y_1_: if (Contraction OR Bleeding OR Threatened Miscarriage) y_1_  = 1 / else y_1_ = 0

y_2_: if (Hypertension OR Pre-Eclampsia) y_1_ = 1 / else y_1_ = 0

y_3_: if (Infection AND Fever) y_3_ = 1 / else y_3_ = 0

Gestational Problem = y_1_ + y_2_ + y_3_ + Gestational Diabetes

if (Gestational Problem >= 3) Gestational Problem = 3

- **Drugs (levels 0, 1 and 2):**

if (Alcohol >= 1) Alcohol = 1

if (Smoke >= 1) Smoke = 1

if (Marijuana >= 1) Marijuana = 1

Drugs = Alcohol + Smoke + Marijuana

if (Drugs >= 2) Drugs = 3

- **House Income (levels 0, 1 and 2):**

if (House Income <= 5) House Income = 0

if (House Income == 6) House Income = 1

if (House Income >= 7) House Income = 2

- **Mother Education (levels 0, 1 and 2):**

if (Mother Education <= 5) Mother Education = 0

if (Mother Education == 6 OR == 7) Mother Education = 1

if (Mother Education >= 8) Mother Education = 2

- **Father Education (levels 0, 1 and 2):**

if (Father Education <= 5) Father Education = 0

if (Father Education == 6 OR == 7) Father Education = 1

if (Father Education >= 8) Father Education = 2

- **Stress (levels 0, 1, 2 and 3): job change, partner losing job, moving home, death of a loved one, problems with justice system, being robbed, witness a crime, live alone and poverty;**

if (Job Change >= 1) Job Change = 1

if (Partner Losing Job >= 1) Partner Losing Job = 1

if (Moving Home >= 1) Moving Home = 1

if (Death of a loved one >= 1) Death of a loved one = 1

if (Problem with Justice System >= 1) Problem with Justice System = 1

if (Being robbed >= 1) Being robbed = 1

if (Witness a Crime >= 1) Witness a Crime = 1

if (Live Alone >= 1) Live Alone = 1

if (Poverty >= 1) Poverty = 1

Stress = Σ (all stress)

if (Stress >= 3) Stress = 3

- **Aggression (levels 0, 1 and 2): physical, psychological, verbal and sexual aggression by partner, family member or others.**

if (Physical by Partner >= 1) Physical by Partner = 1

if (Physical by Familiar >= 1) Physical by Family Member = 1

if (Physical by Others >= 1) Physical by Others = 1

if (Psychological by Partner >= 1) Psychological by Partner = 1

if (Psychological by Family Member >= 1) Psychological by Family Member = 1

if (Psychological by Others >= 1) Psychological by Others = 1

if (Verbal by Partner >= 1) Verbal by Partner = 1

if (Verbal by Family Member >= 1) Verbal by Family Member = 1

if (Verbal by Others >= 1) Verbal by Others = 1

if (Sexual by Partner >= 1) Sexual by Partner = 1

if (Sexual by Family Member >= 1) Sexual by Family Member = 1

if (Sexual by Others >= 1) Sexual by Others = 1

Aggression = Σ (all aggressions)

if (Aggression == 1 OR == 2) Aggression = 1

if (Aggression >= 3) Aggression = 2

**Supplemental Figures and Tables**

**
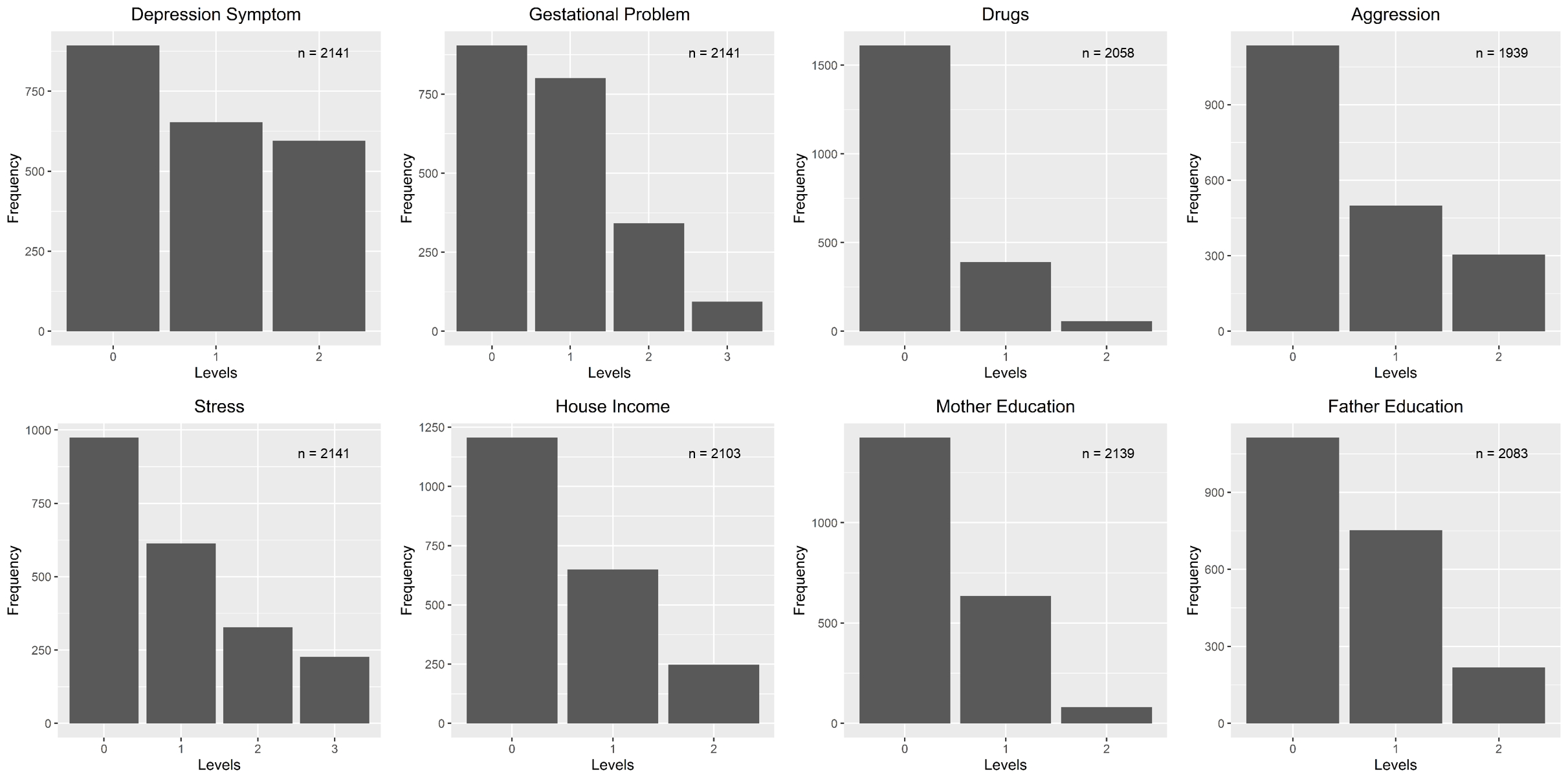
**

**Supplemental Figure 1 - Risk Factors Distribution.** Distribution of response levels for risk factors variables. The numbers of individuals (n) are displayed in the respective graphs.

**
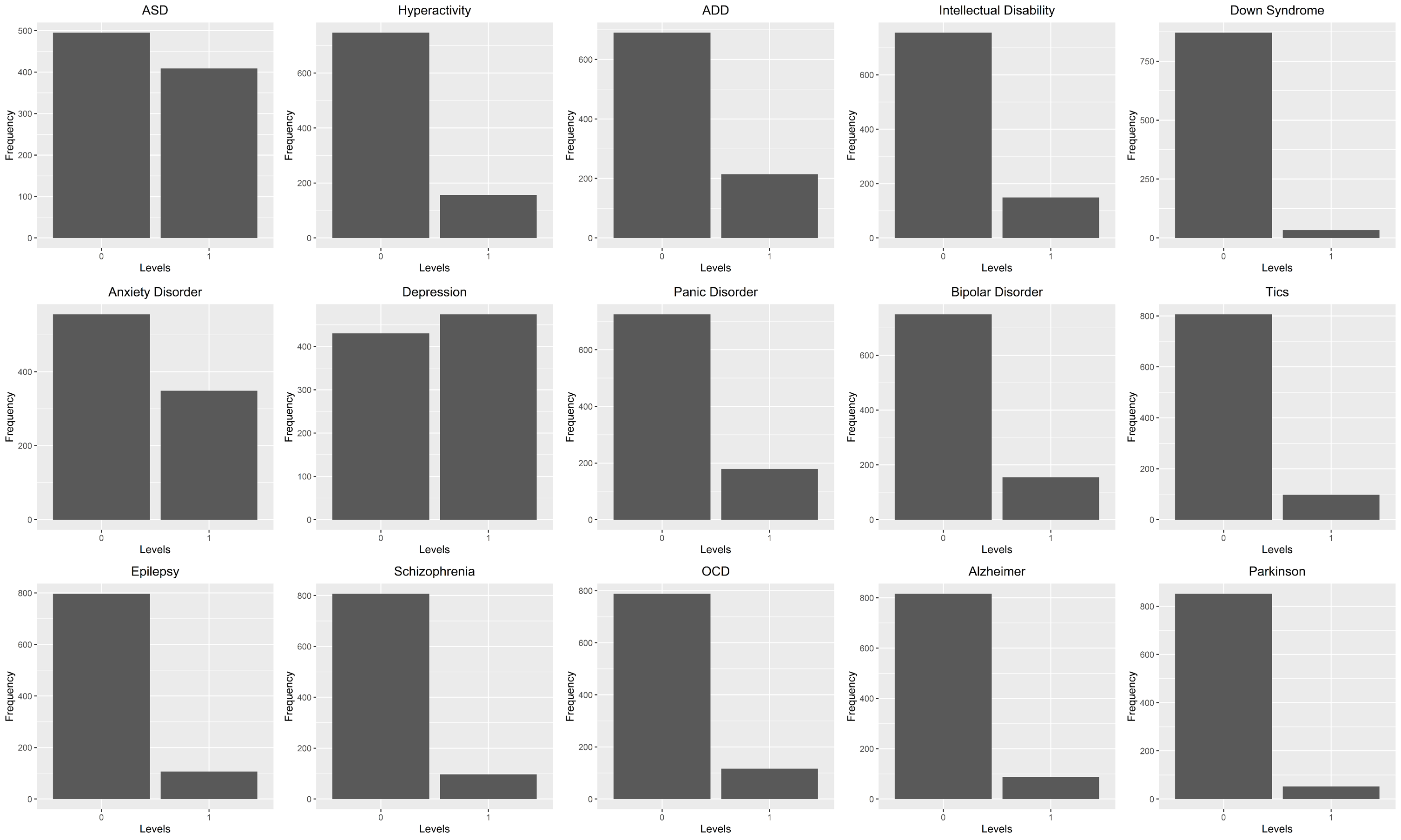
**

**Supplemental Figure 2 - Family History Distribution.** Distribution of response levels for family history variables. In all graphs, the number of individuals (n) was 904. Autism Spectrum Disorder (ASD); Attention Deficit Disorder (ADD); Obsessive Compulsive Disorder (OCD).

**
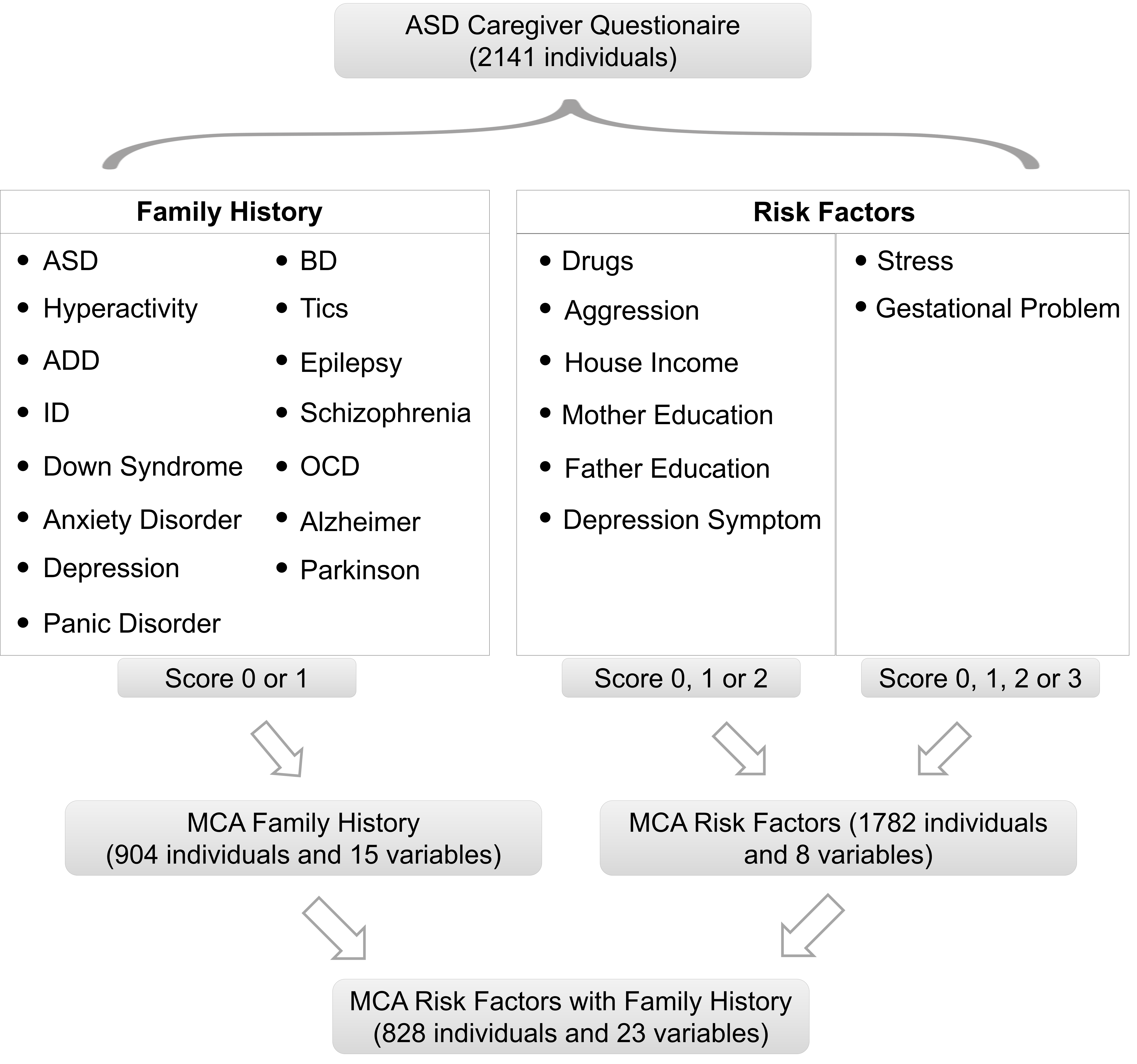
Supplemental Figure 3 - Flowchart.** Flowchart of the methodological approach. Autism Spectrum Disorder (ASD); Bipolar Disorder (BD); Attention Deficit Disorder (ADD); Intellectual Disability (ID); Obsessive Compulsive Disorder (OCD).

**

**

**B**

**A**

**

**

**

**

**C**

**Supplemental Figure 4 - MCA with risk factors and family history variables (828 individuals and 23 variables).** A, Barplot of contribution of variables to first dimension; B, Barplot of contribution of variables to second dimension; C, MCA biplot visualization of response levels. Autism Spectrum Disorder (ASD); Bipolar Disorder (BD); Attention Deficit Disorder (ADD); Obsessive Compulsive Disorder (OCD); Intellectual Disability (ID).

**

**

**Supplemental Figure 5 - MCA biplot of individuals by sex considering EF and FH variables.** Males and females are represented by blue and red, respectively. Abbreviations: ASD: Autism Spectrum Disorder; BD, Bipolar Disorder; ADD, Attention Deficit Disorder; OCD, Obsessive Compulsive Disorder; ID, Intellectual Disability.

**Supplemental Tables**

| Label | Number of questions | Levels | Content of the questions | Dismissed answers |
| --- | --- | --- | --- | --- |
| Depression Symptom | 07 | 03 | Sadness, Anxiety, Weeping, Demotivation, Irritability, Guilty, Suicidal Ideation | 0 |
| Gestational Problem | 07 | 04 | Contraction, Bleeding, Threatened Miscarriage, Hypertension, Pre-Eclampsia, Infection AND Fever | 0 |
| Drugs | 03 | 03 | Alcohol, Smoke, Marijuana | 83 |
| House Income | 01 | 03 | House Income | 37 |
| Mother Education | 01 | 03 | Mother Education | 01 |
| Father Education | 01 | 03 | Father Education | 57 |
| Stress | 09 | 03 | Job change, partner losing job, moving home, death of a loved one, problems with justice system, being robbed, witness a crime, live alone and poverty | 0 |
| Aggression | 12 | 03 | Physical, psychological, verbal and sexual aggression by partner, family member or others. | 202 |
| Total | | | | 380* |

*Some respondents have answered "I don't know" for more than one question.

**Supplemental Table 1** - Number of questions and the content of different questions used to compose different environmental exposure scores and dismissed individuals.

**
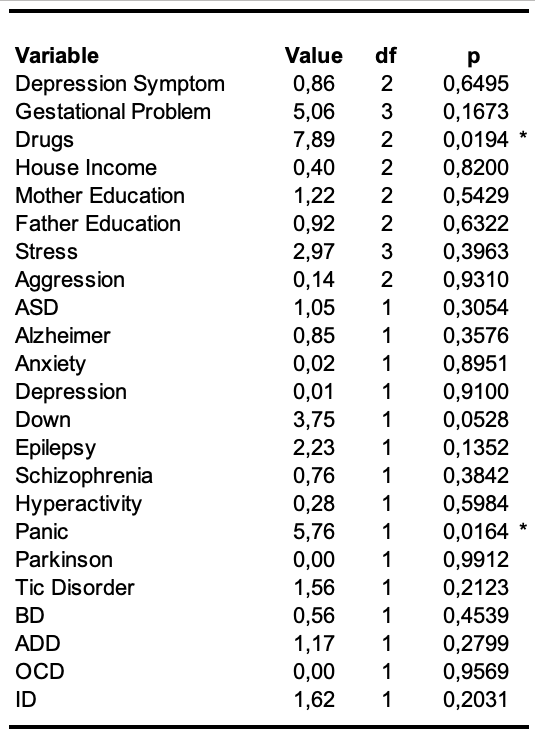
**

**Supplemental** **Table 2 - Gestational environmental and FH scores between males and females.** Results of the association test between biological sex versus environmental factors and Family History variables. Abbreviations: ASD: Autism Spectrum Disorder; BD, Bipolar Disorder; ADD, Attention Deficit Disorder; OCD, Obsessive Compulsive Disorder; ID, Intellectual Disability.

**
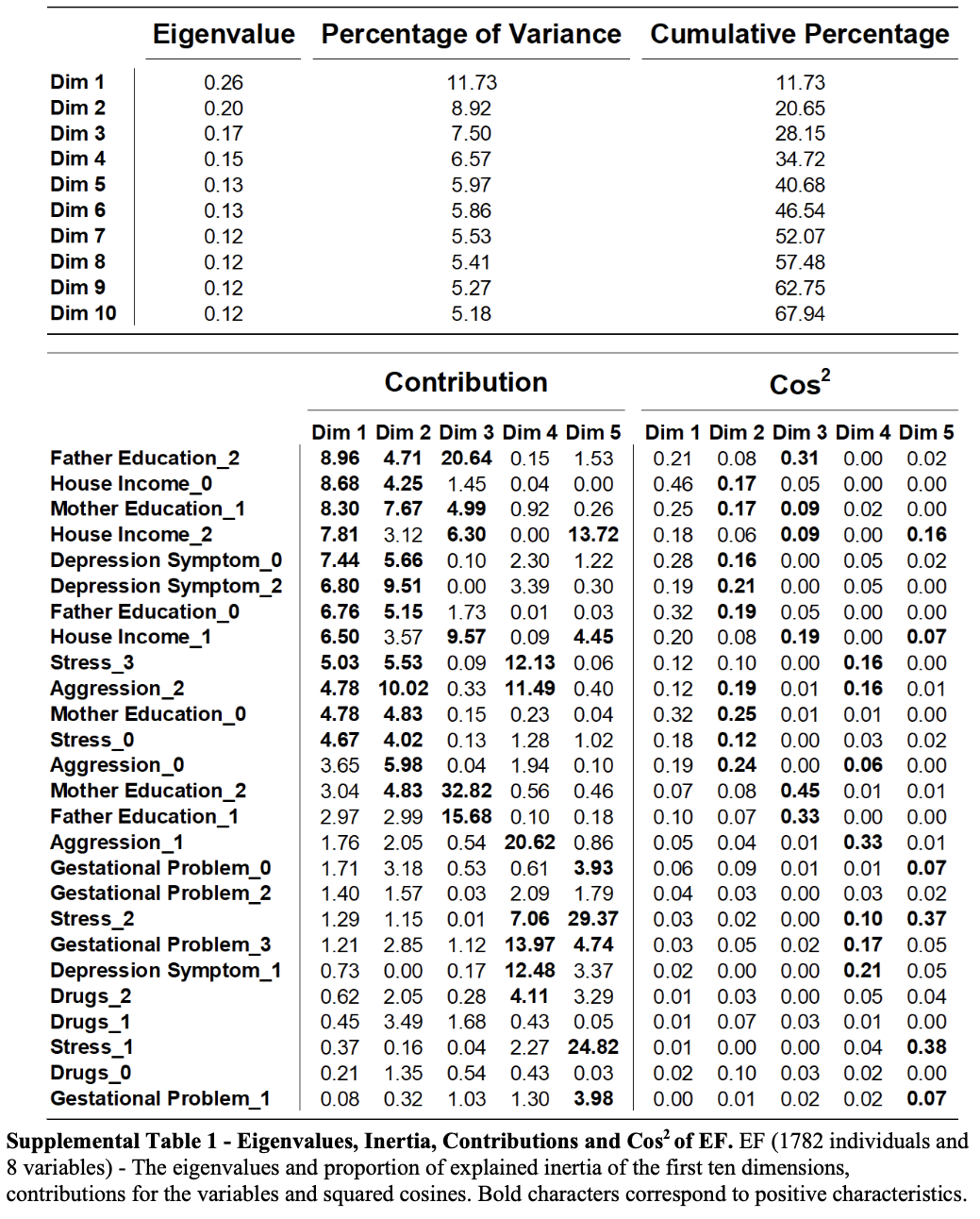
**

**Supplemental** **Table 3 - Eigenvalues, Inertia, Contributions and Cos^2^ of Risk Factors.** Risk factors (1782 individuals and 8 variables) - The eigenvalues and proportion of explained inertia of the first ten dimensions, contributions for the variables and squared cosines. Bold characters correspond to positive characteristics.

**
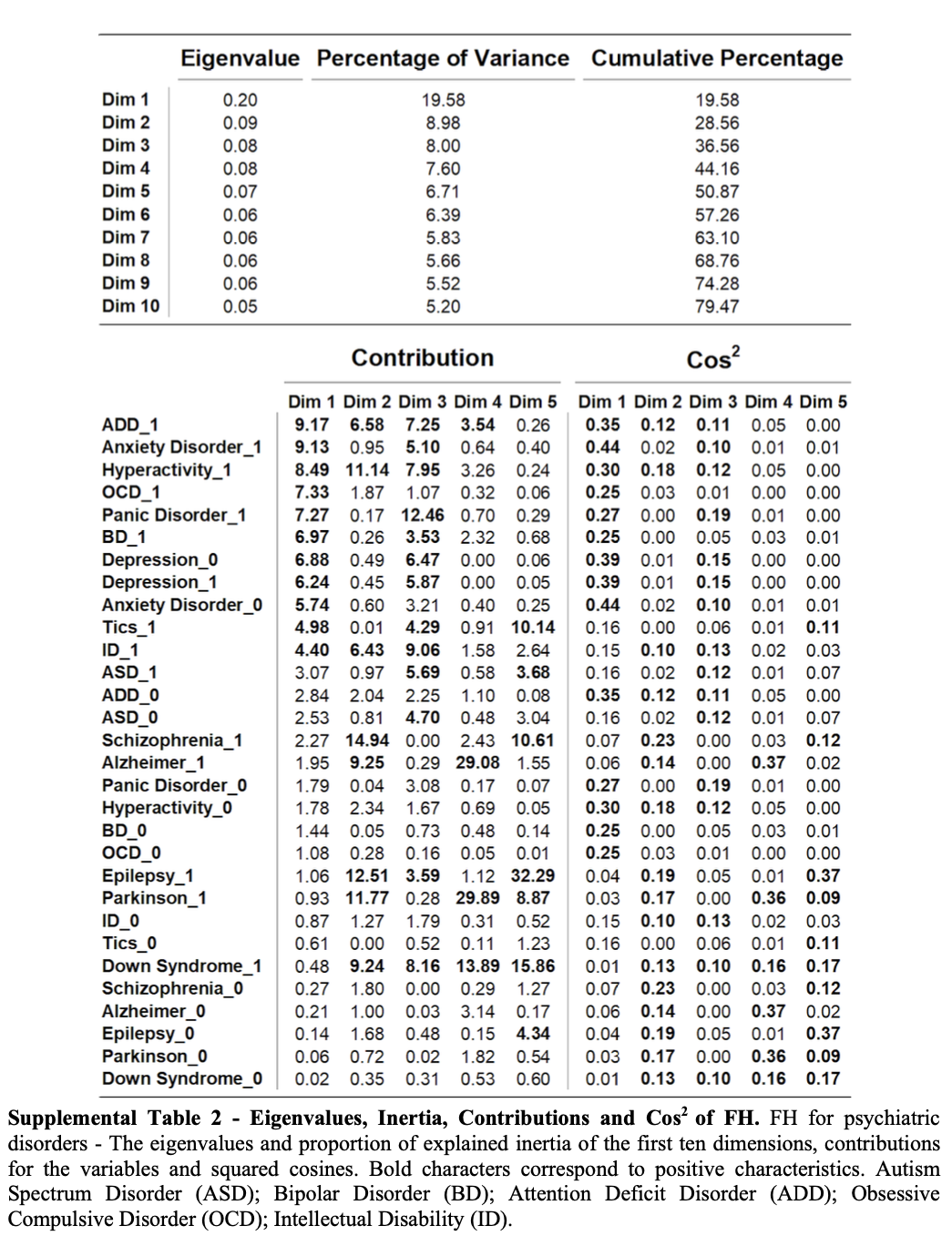
**

**Supplemental** **Table 4 - Eigenvalues, Inertia, Contributions and Cos^2^ of Family History.** Family history for psychiatric disorders - The eigenvalues and proportion of explained inertia of the first ten dimensions, contributions for the variables and squared cosines. Bold characters correspond to positive characteristics. Autism Spectrum Disorder (ASD); Bipolar Disorder (BD); Attention Deficit Disorder (ADD); Obsessive Compulsive Disorder (OCD); Intellectual Disability (ID).


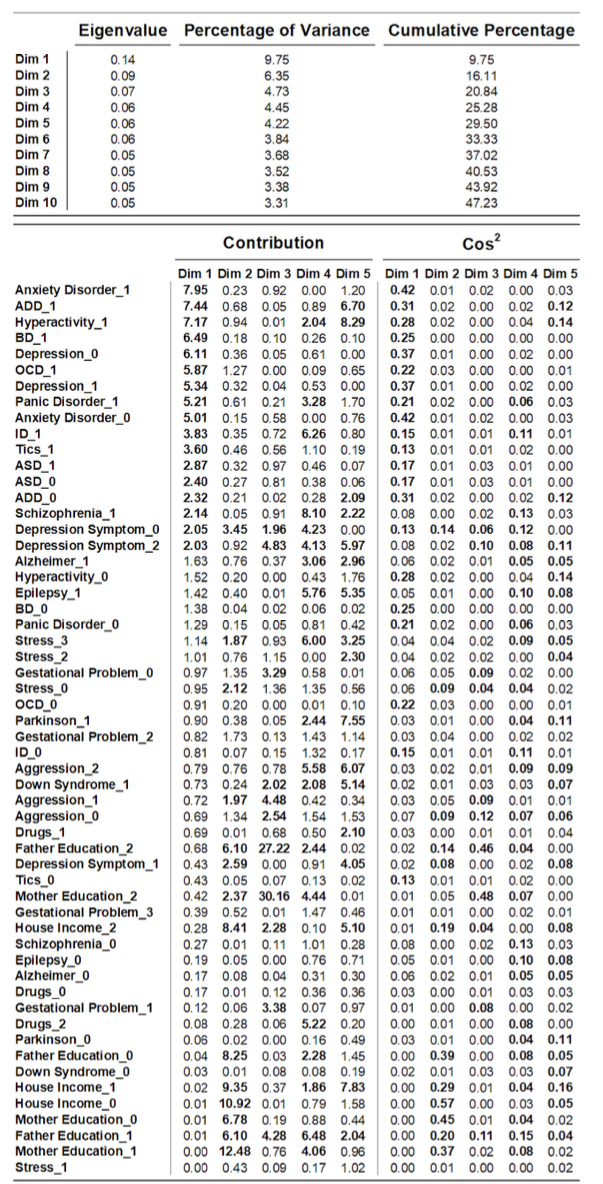


**Supplemental** **Table 5 - Eigenvalues, Inertia, Contributions and Cos^2^ of Risk Factors and Family History.** Risk factors and family history for psychiatric disorders - The eigenvalues and proportion of explained inertia of the first ten dimensions, contributions for the variables and squared cosines. Bold characters correspond to positive characteristics. Autism Spectrum Disorder (ASD); Bipolar Disorder (BD); Attention Deficit Disorder (ADD); Obsessive Compulsive Disorder (OCD); Intellectual Disability (ID).
